# Mental Fatigue Detection of Crane Operators Based on Electroencephalogram Signals Acquired by a Novel Rotary Switch-Type Semi-Dry Electrode Using Multifractal Detrend Fluctuation Analysis

**DOI:** 10.3390/s25133994

**Published:** 2025-06-26

**Authors:** Fuwang Wang, Daping Chen, Xiaolei Zhang

**Affiliations:** 1 School of Mechanic Engineering, Northeast Electric Power University, Jilin 132012, Chinatingyusheng2022@163.com (D.C.); 2 Gongqing Institute of Science and Technology, Jiujiang 332020, China

**Keywords:** rotary switch type, semi-dry electrode, crane operator, EEG, MF-DFA, mental fatigue

## Abstract

The mental fatigue of crane operators can pose a serious threat to construction safety. To enhance the safety of crane operations on construction sites, this study proposes a rotary switch semi-dry electrode for detecting the mental fatigue of crane operators. This rotary switch semi-dry electrode overcomes the problems of the large impedance value of traditional dry electrodes, the cumbersome wet electrode operation, and the uncontrollable outflow of conductive liquid from traditional semi-dry electrodes. By designing a rotary switch structure inside the electrode, it allows the electrode to be turned on and used in motion, which greatly improves the efficiency of using the conductive fluid and prolongs the electrode’s use time. A conductive sponge was used at the electrode’s contact end with the skin, improving comfort and making it suitable for long-term wear. In addition, in this study, the multifractal detrend fluctuation analysis (MF-DFA) method was used to detect the mental fatigue state of crane operators. The results indicate that the MF-DFA is more responsive to the tiredness traits of individuals than conventional fatigue detection methods. The proposed rotary switch semi-dry electrode can quickly and accurately detect the mental fatigue of crane operators, provide support for timely warning or intervention, and effectively reduce the risk of accidents at construction sites, enhancing construction safety and efficiency.

## 1. Introduction

With the continuous progress of social industrialization, cranes play a vital role in construction, manufacturing, logistics, and other fields. However, this is accompanied by crane operator mental fatigue caused by safety risks and production efficiency decline. For crane operators, mental fatigue may lead to decreased attention to the surrounding environment, slow reaction speed, and thus increase the risk of accidents [1]. Therefore, fatigue detection for crane operators is important for improving construction safety.

In order to effectively detect mental fatigue in humans, many methods have been proposed in domestic and international studies. Current methods for detecting mental fatigue include subjective questionnaires [2], facial detection [3], and eye movement characteristics [4]. However, these methods have limitations, such as subjective questionnaire assessment that relies on individual subjective reports and may vary depending on subjective perception. Facial detection may result in a misjudgment of fatigue status due to a variety of factors, such as light or wearing sunglasses or masks. Eye movement characteristics are susceptible to interference from the external environment, such as bright light, reflections, vibrations, etc., which may affect the driver’s eye movement activities, thus affecting the judgment of fatigue status. Over the past few years, with the advancement of electroencephalography (EEG) signal processing technology and the development of wearable sensors, more and more researchers are using EEG signals to detect mental fatigue. As a physiological signal that directly reflects brain activity, the EEG signal has high temporal resolution and sensitivity, which can provide more accurate and objective data support for mental fatigue monitoring [5]. Therefore, in this paper, the EEG signals of operators were used to characterize their mental fatigue changes.

The three primary types of EEG signal acquisition sensors are semi-dry, dry, and wet electrodes. The benefits of wet electrodes include stability, a high signal-to-noise ratio, low impedance, and a dependable signal [6]. Wang et al. proposed a new nanoclay-enhanced hydrogel wet electrode. The results showed that this electrode can be applied to wearable monitors, which can acquire human EEG signals highly sensitively and stably over a relatively long period of time [7]. However, the drawbacks of this wet electrode, such as inconvenient handling, poor comfort, and short duration of continuous signal acquisition, limit its application in wearable daily ambulatory EEG monitoring. Dry electrodes are more convenient than traditional electrodes as they do not need conductive paste. Wang et al. proposed a pyramid-type microneedle dry electrode. EEG data were captured directly with three pairs of typical wet needle/microneedle electrodes. To evaluate signal quality, the power spectral density of wet needle and microneedle electrodes was compared. The study found that the microneedle dry electrode is suitable for use as a biopotential electrode to acquire EEG signals due to its low skin electrode contact impedance [8]. However, the microneedle dry electrode is an invasive dry electrode, which is complicated and costly to manufacture, and the tip of the electrode is fragile and prone to skin infection. Chen et al. introduced a non-invasive dry electrode with adaptive mechanical design to address the limitations of invasive dry electrodes for measuring EEG signals in the hairy region of the brain. This study demonstrated that the suggested non-contact dry electrode effectively captured EEG signals in hairy areas and minimized the impact of motion artifacts [9]. However, this dry electrode has a high electrode impedance due to the absence of a conductive gel, resulting in poor quality of the acquired EEG signal, and the use of rigid conductive materials may lead to discomfort for the subject [10]. To address the limitations of traditional wet and dry electrodes, researchers have proposed various types of semi-dry electrodes that avoid soiling the hair, prevent short-circuiting, and are capable of performing the localized replenishment of conductive fluids, which reduces the impedance of the electrodes to the scalp [11]. Pasion et al. introduced a polymer core-based semi-dry electrode that combines traditional Ag/AgCl electrodes and polymer core electrodes for EEG recording. The outcomes show that the performance of the polymer core electrode is comparable to that of the conventional Ag/AgCl electrode [12]. Li et al. introduced a flexible semi-dry electrode with multiple claws and channels for capturing EEG signals. The electrode reduced contact resistance with the scalp by utilizing a well-adhered internal carbon nanotube conductive network and a flexible Cu-TiO2-CNT@PDMS substrate. This study discovered that there was a correlation coefficient of more than 99.3% between the semi-dry electrode and the spontaneous EEG signal produced by commercial Ag/AgCl synchronization [13]. Hua et al. developed a flexible multilayer semi-dry electrode for EEG acquisition. The results demonstrated that the electrode could be used well for EEG monitoring in dense scalp locations [14]. However, current semi-dry electrodes also generally share a common problem. None of them can realize the function of use-as-you-go and turn on-as-you-go. When it is necessary to interrupt the experiment, the semi-dry electrode must be removed to stop acquiring signals, which makes the operation process cumbersome.

In view of the above problems, this study proposes a rotary switch EEG semi-dry electrode. By designing a knob switch structure inside the electrode, the electrode can be opened and closed at any time, which is convenient for users to perform EEG signal acquisition when needed, enhancing its portability and usability. In addition, the use of conductive sponges instead of traditional rigid electrode cores can improve the comfort of the subjects. The conductive sponge is soft and breathable, better suited for the scalp curve, reduces discomfort during use, and is comfortable for long-term wear. Due to the ready-to-use function and comfortable wearing experience, this designed EEG semi-dry electrode is more suitable for mobile EEG acquisition scenarios, such as outdoor experiments, mobile applications, or long-term monitoring.

Mental fatigue detection methods often used by researchers include power spectrum [15], sample entropy (SampEn) [16], Lempel-Ziv (LZ) complexity [17], and so on. However, these traditional methods have some limitations. For example, SampEn methods are extremely sensitive to noise and interference in the data. Power spectrum methods assume that the signal is smooth, but EEG signals are usually nonsmooth (or noisy), which may lead to distorted or inaccurate processing results. In addition, the results of LZ complexity are affected by the length of the signal, and shorter signals may lead to inaccurate complexity estimates. These challenges in handling noisy, non-stationary, and finite-length signals are not unique to EEG analysis; they also arise in complex mechanical system monitoring, such as in the assessment of high-speed train bearing health using acoustic emission (AE) techniques [18]. Addressing the dynamic and transient nature of signals, especially those indicative of incipient faults, often requires adaptive approaches. For instance, adaptive step-size front–back tracking algorithms have been developed to improve the precision of capturing localized events and transient features in AE signals [19]. Furthermore, understanding the fundamental mechanisms is crucial; dynamic detection mechanism models for AE signals have been established specifically for high-speed train axle box bearings with localized defects, elucidating the relationship between defect characteristics and emitted AE signatures [20,21]. Considering the limitations of conventional approaches and inspired by advancements in adaptive signal processing for mechanical diagnostics, this study adopted the multifractal detrended fluctuation analysis (MF-DFA) method to detect the mental fatigue status of crane operators [22]. The MF-DFA method has good analytical processing ability when dealing with one-dimensional time series signals and can effectively analyze the multiple fractal features of nonlinear and nonsmooth signals, with low data dimensionality requirements.

## 2. Materials and Methods

### 2.1. Rotating Switch-Type Semi-Dry Electrode

#### 2.1.1. Structural Design

**Figure 1:**
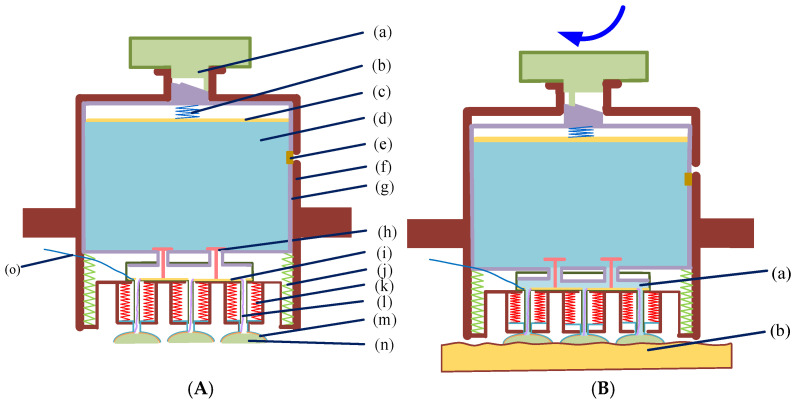
The structure of the rotary switch-type semi-dry electrode developed for this research. (**A**) Schematic illustration of the electrode’s cross-sectional structure when it is not in operation. (**a**) Rotary switch; (**b**) spring 1; (**c**) push plate; (**d**) PVA sponge; (**e**) stopper; (**f**) electrode housing; (**g**) housing for storing the PVA sponge; (**h**) valve; (**i**) copper plate 1; (**j**) spring 2; (**k**) spring 3; (**l**) conductor; (**m**) copper sheet; (**n**) electrically conductive sponge; (**o**) signaling wire. (**B**) Schematic structure of the electrode cross-section in operation. (**a**) Conductive liquid; (**b**) skin.

Figure 1 depicts the structure of the rotary switch-type semi-dry electrode designed for this study.

The main components of the rotary switch-type semi-dry electrode proposed in this study include rotary switch, spring 1, push plate, PVA sponge (Changzhou Ruixuan Purification Technology Co., Ltd., Changzhou, China), stopper, electrode housing, valve, copper plate (Dongguan Jiashan Copper Materials Co., Ltd., Dongguan, China), spring 2 (Hangzhou Yuanfang Spring Factory, Hangzhou, China), spring 3, hemispherical housing, conductive sponge (Shenzhen Hui Ruhai Technology Co., Ltd., Shenzhen, China), and so on. The function of the rotary switch is to control whether the valve is open. When the switch is open, the valve opens and the conductive liquid (Suzhou Naplusite Nano Materials Co., Ltd., Suzhou, China) flows out; when the switch is closed, the valve closes and the conductive liquid stops flowing. Spring 1 squeezes the conductive liquid held in the PVA sponge so that when the valve is opened, the conductive liquid can flow downward into the conductive sponge. The PVA sponge stores the conductive liquid. The sealing plug prevents the conductive liquid from overflowing. The PVA sponge shell protects the PVA sponge. The valve works with the rotary switch to control the flow of the conductive liquid. The copper plate collects the current. Spring 2 provides upward force to the PVA sponge housing, causing the valve to close. The wire pools the current collected by copper plate 1. Copper plate 2 also pools the current. The conductive sponge collects EEG signals. A threaded connection is used between the electrode head and the hemispherical housing, making it easier to replace the conductive sponge. Additionally, the conductive sponge material is a polyurethane material prepared by foaming technology and subsequently treated with chemical nickel plating, imparting excellent conductivity and corrosion resistance. The material has a surface resistance of about 100 Ω/m^2^ and a vertical resistance of about 5 Ω, indicating excellent conductive properties. Furthermore, the diameter of the metal particles in the surface coating of the material is less than 700 nanometers, further ensuring its conductivity and stability.

The working process of the semi-dry electrode: First, open the sealing plug and inject the conducting liquid into the PVA sponge using a straw. Then, close the sealing plug. The valve is opened by rotating the switch at the end of the electrode. At this point, the conductive liquid in the sponge flows downwards due to the action of spring, entering the conductive sponge and then contacting the skin.

#### 2.1.2. Manufacturing of Electrodes

**Figure 2:**
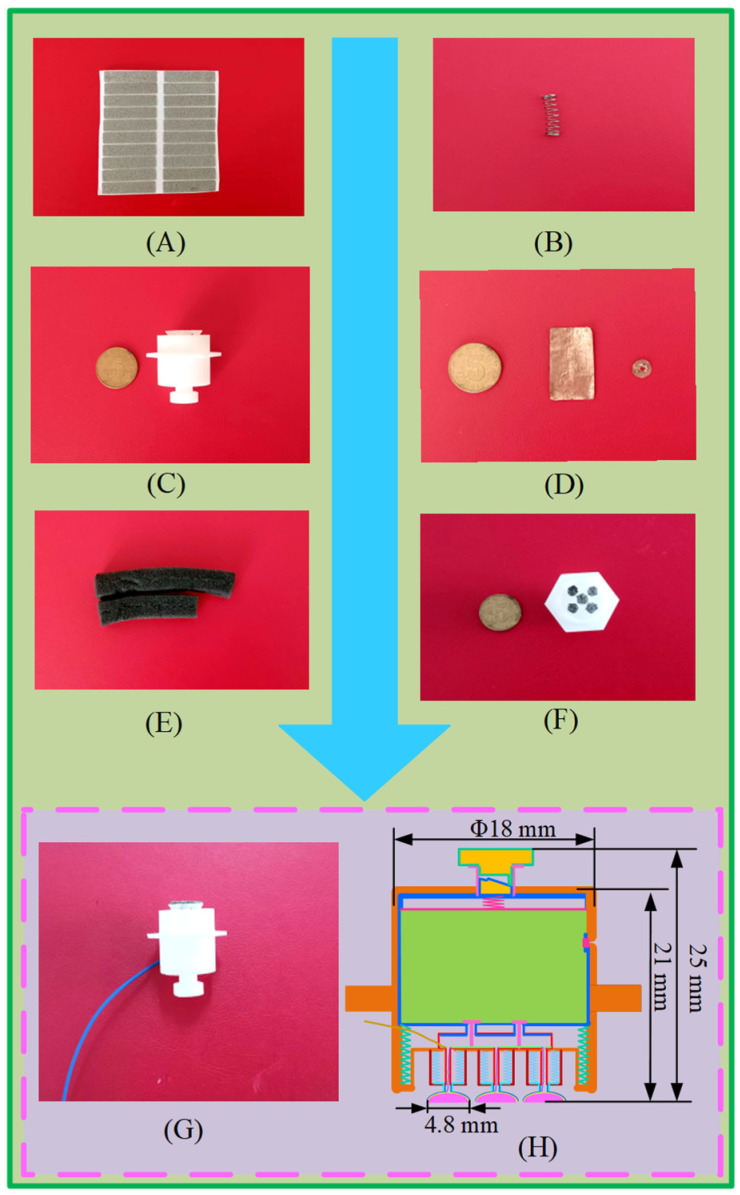
The fabrication procedure for the rotary switch-type semi-dry electrode proposed in this work. (**A**) Conductive sponge; (**B**) spring; (**C**) electrode housing fabricated by 3D printing; (**D**) copper sheet; (**E**) PVA sponge; (**F**) top view after encapsulating the sponge into the electrode; (**G**) fabricated electrode; (**H**) dimensions of the electrode.

Figure 2 displays the fabrication procedure of the electrodes created in this work.

The production steps of the semi-dry electrode are as follows: draw the main parts of the electrode using 3D drawing software (Solidworks 2021) and then use a 3D printer to print the drawn model to create the electrode shell and other components. The spring, PVA sponge, valve, copper plate, and wire are installed into the electrode assembly. Next, a rectangular copper sheet is cut into a round shape with a small hole in the center and then glued inside the hemispherical shell. Finally, a conductive sponge is glued to the copper sheet.

#### 2.1.3. Impedance Test of Electrode

**Figure 3:**
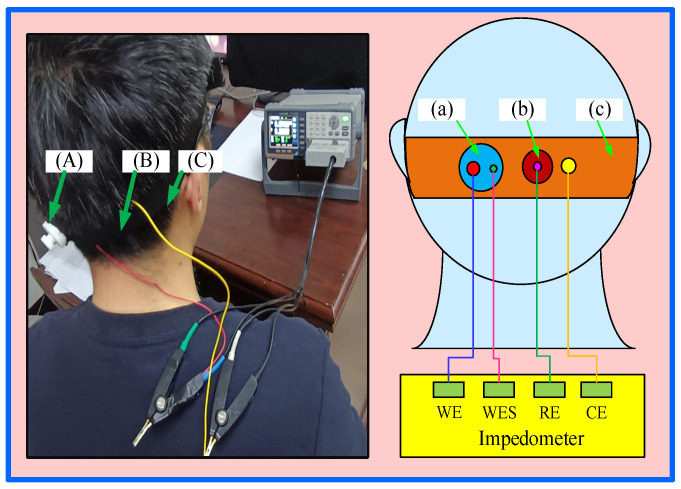
Diagrammatic representation of the electrode contact impedance test. (**A**) electrode; (**B**) Ag/AgCl wet electrode; (**C**) bandage; (**a**) schematic diagram of electrode; (**b**) diagram schematic for the Ag/AgCl wet electrode; (**c**) schematic diagram of bandage.

One important factor influencing how well an electrode functions when in use is the contact impedance between the electrode and the skin. A lower contact impedance reduces the noise generated during use and thus contributes to a higher-quality EEG signal [23]. Other items that need to be used in the measurement of electrode impedance experiments include abrasive cleaner, conductive paste, traditional Ag/AgCl electrodes, syringes, and bandages. In this study, the Ag/AgCl electrode was chosen as the reference electrode. Before the experiment, an appropriate amount of abrasive paste was gently applied to the O1 channel on the occipital region of the subject’s scalp with a dust-free cloth, and conductive cream was then applied. The distance between the semi-dry and wet electrodes proposed in this study was set to be 2 cm. The semi-dry and wet electrodes were attached to the scalp using an elastic bandage. The circuit diagram for the electrode–skin contact impedance test is shown in Figure 3.

This experiment used an LCR 6300 impedance analyzer (Suzhou Yudeng Technology Co., Ltd., Suzhou, China) to measure frequencies ranging from 10 Hz to 1000 Hz in 1 Hz increments. In this study, the contact impedance was measured using the dual-electrode method. The working electrode (WE) and working electrode sensor (WES) were connected to the two silver-plated terminals of the semi-dry electrode, while the counter electrode (CE) was connected to an elastic band made of pure copper fabric. Finally, the reference electrode (RE) was connected to a Ag/AgCl electrode.

#### 2.1.4. Electrode Life Test

**Figure 4:**
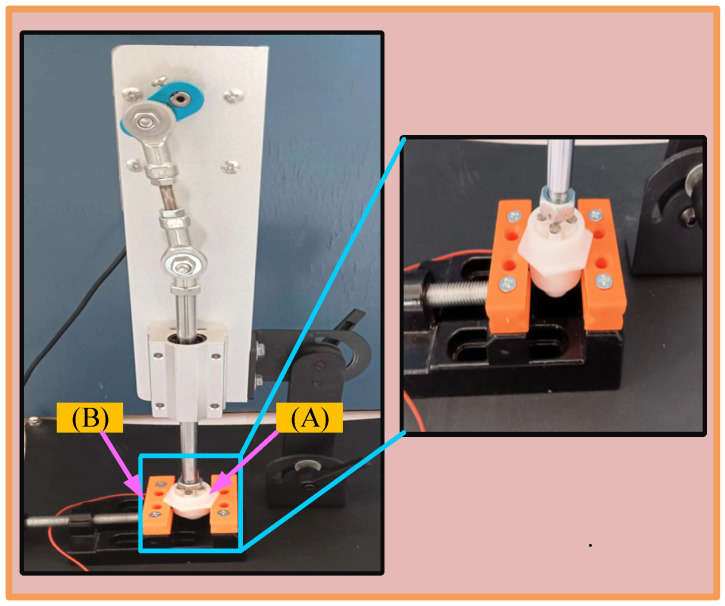
Test setup for service life testing of electrodes. (**A**) Fixture; (**B**) electrode.

The experiment aimed to assess the electrodes’ long-term mechanical stability through repetitive compression. The testing device is manufactured by Shengda Machinery Manufacturing Co., Ltd. in Dongguan, China, and is powered by the ZK-DIY-555 motor. This motor has excellent performance specifications, with an idle speed range of 2 to 1000 revolutions per minute, an idle current of 0.08 to 0.15 amps, and a rated power of 15 watts, which can meet the experimental requirements for the precise control of speed and current. The device’s telescoping rod is designed for optimal functionality, with a travel range of 0 to 3.5 cm, enabling the precise control of the pressure and contact force applied by the electrodes. The motor’s operating voltage can be selected as either 12 V or 24 V to accommodate different experimental needs. During the experiment, the compression device pressed down on the electrode 100 times per minute, and the entire experiment lasted for a total of approximately 90 min. The experiment was divided into 7 stages, and at the end of each stage, the staff accurately measured the height and elasticity of the electrode. Through these measurements, it is possible to assess changes in electrode performance during multiple compression cycles, thereby providing data to optimize electrode design and extend their service life. Figure 4 shows the experimental setup.

### 2.2. Experiment

#### 2.2.1. Subjects

This study recruited 12 people, including 10 males and 2 females, aged 30 ± 4.5 years (SD). All subjects were trained in topics related to construction site precautions. To ensure the accuracy of the experimental results, the subjects were asked to get enough sleep on the day of the experiment and avoid staying up late, consuming alcohol, taking drugs, or engaging in strenuous exercise. In addition, before the start of the experiment, subjects were assigned a number labeled “A” to “L”. All subjects were required to fully understand the purpose and procedure of the trial and sign an informed consent form before proceeding. The study protocol was authorized by the Northeast Electric Power University Hospital following the ethical principles of the World Medical Association (Declaration of Helsinki).

#### 2.2.2. Experimental Paradigm

**Figure 5:**
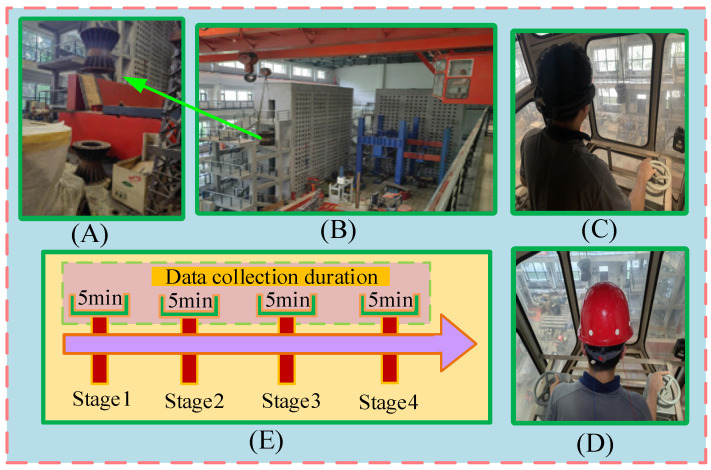
Flowchart of the experiment. (**A**) Prefabricated parts; (**B**) operator-driven crane; (**C**) Emotiv device; (**D**) electrodes proposed in this study; (**E**) diagram of EEG data acquisition program.

The experiment was conducted in an actual building setting between 14:00 and 17:00. Four acquisition stages were used to collect the EEG data: stage 1 (14:00–14:05), stage 2 (14:05–14:30), stage 3 (14:30–15:00), and stage 4 (15:00–15:05). The signals were collected at 30 min intervals. Every participant performed two experiments: one with an Emotiv electrode and the other with a semi-dry electrode. Both electrodes were positioned using an international electrode system of 10–20 and sampled at 128 Hz. Before the experiment started, the experimenter introduced the purpose, procedure, and operation of the experiment to the participants. In this study, in order to accurately synchronize subjective fatigue status with physiological signals, we asked drivers to complete the Karolinska Sleepiness Scale (KSS) self-assessment within the first five minutes of continuous EEG signal collection [24]. The experimental setup is shown in Figure 5.

### 2.3. Methods

#### 2.3.1. Data Preparation Algorithm

Compressive sensing (CS) theory suggests that when a signal is sparse or compressible, it can be accurately or approximately reconstructed by collecting a small number of signal projection values [25]. The introduction of CS theory has made it possible to collect signals at rates far below those required by the Nyquist sampling theorem while ensuring that no information is lost, and to completely restore the signal, thereby transforming signal sampling into information sampling. This study uses CS to preprocess EEG signals to reduce training time.

#### 2.3.2. MF-DFA Algorithm

MF-DFA is a method used for analyzing signal dynamics based on DFA combined with multiple fractal spectra. The detailed theoretical content is elaborated as follows [26]:

Given a time series *z* (*k* = 1, 2,…, *N*), the computation using the MF-DFA method is divided into the steps listed below.

(1) Construct the signal profile *Y*(*i*) as shown in Equation (1).
  (1) $$Y(i)=\sum_{k=1}^{i}\left[z_{k}-\langle z\rangle \right],i=1,\ldots ,N;\langle z\rangle =\frac{1}{N}\sum_{k=1}^{N}z_{k}$$
(2) Divide contour $Y\left(i\right)$ into $N_{s}=\mathrm{int}\left(N_{s}\right)\;$ non-overlapping data segments of equal length s.
(3) Separate contour $Y\left(i\right)$ into $N_{s}=\mathrm{int}\left(N_{s}\right)\;$ data segments of equal lengths that do not overlap.
(4) Calculate the local trend function $y_{v}(i)$ by fitting a regression based on an m-order polynomial to the data in subinterval $v\left(v=1,2,\ldots ,N_{s}\right)$.
(5) Calculate the data variance for each segment as shown in Equations (2) and (3).
  (2) $$F^{2}(v,s)=\sum_{i=1}^{s}{\left\{Y[|v-1|s+i]-y_{v}(i)\right\}}^{2},v=1,2,\ldots ,N_{s}\;$$
  (3) $$F^{2}(v,s)=\sum_{i=1}^{s}{\left\{Y\left[N-\left(v-N_{s}\right)s+i\right]-y_{v}(i)\right\}}^{2},v=N_{s}+1,N_{s}+2,\ldots ,2N_{s}$$
(6) Calculate the mean value of the *q*th order fluctuation function as indicated in Equation (4).
  (4) $$F_{q}(s)={\left\{\frac{1}{2N_{s}}\sum_{v=1}^{2N_{s}}{\left[F^{2}(v,s)\right]}^{\frac{q}{2}}\right\}}^{\frac{1}{q}}$$
(7) The existence of a self-similarity characteristic in the time series *z_k_* is indicated by a power law relationship between the *q*th order fluctuation function mean and the time scale *s*, as shown in Equation (5).
  (5) $$F_{q}(s)\sim s^{H(q)}$$

H(q) is the multifractal scaling index or Hurst exponent, and H(q) varies with *q* if $z_{k}$ is characterized by multifractality; conversely, H(q) is a constant.

Within multifractals, there exists a correlation between the scalar index and the Hurst exponent as depicted in Equation (6).

(6) τ(q)=qH(q)−1

According to the Legendre transform, the singularity index α and the multifractal spectrum f(α) of the sequence $z_{k}(k=1,2,\ldots ,N)$ can be calculated as shown in Equation (7):

(7) $$\left\{\begin{array}{l} \alpha =H(q)+qH^{\prime }(q)\\ f(\alpha )=q\alpha -\tau (q)=q[\alpha -H(q)]+1 \end{array}\right.$$

From the results of the above process, a chart can be drawn for analyzing the multifractal spectrogram of the time series. The drawn image should be a single-peak convex curve. According to the characteristics of the drawn curve, we can extract the features of the multifractal spectrogram and depict the local singularity. The multifractal spectral width illustrates both the signal’s multifractal properties and the actual variation in intensity. The fractal properties of the signal are stronger when the value of the spectral width is larger, indicating stronger signal energy fluctuations and more intense signal variation. Conversely, the more stable the signal is, the fewer fractal characteristics it possesses.

## 3. Results

### 3.1. Electrode Properties

The electrode’s service life and the contact impedance value between it and the skin are the two primary indices influencing its performance. As a result, this study’s contact impedance experiment and electrode service life test were conducted, and the experimental findings are as follows:

#### 3.1.1. Contact Impedance of Electrodes

**Figure 6:**
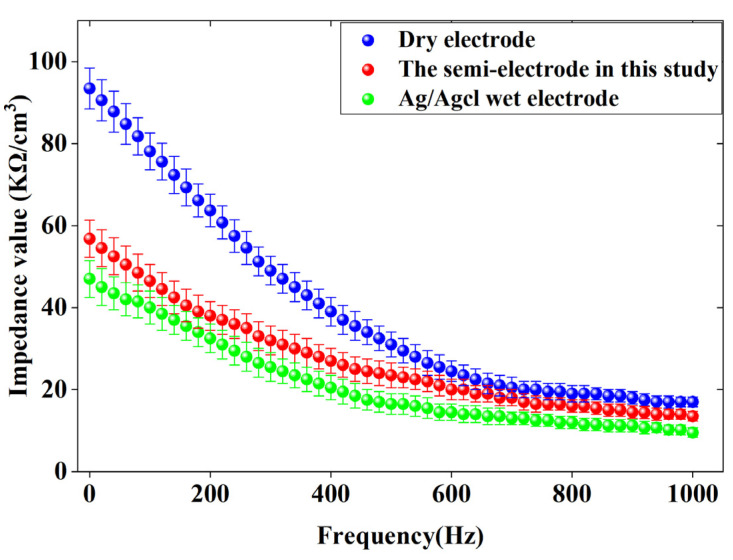
Contact impedance curves between three types of electrodes and skin.

The contact impedance curves between the three electrodes and the skin are shown in Figure 6.

The dry electrode has the highest contact impedance value, as illustrated in Figure 6. The lowest contact impedance is observed in the wet electrode, while the contact impedance of the semi-dry electrode falls between that of the dry and wet electrodes. The high impedance of the dry electrode is primarily due to the absence of an electrolyte at the interface between the electrode and the skin, relying solely on small amounts of perspiration and water as electrolytes. This insufficient electrolyte makes it difficult to establish a stable interface between the electrode and the skin, leading to high impedance due to the skin surface [27]. The conductive liquid or paste used in semi-dry and wet electrodes can quickly create an electrolyte channel at the electrode–skin interface, which explains their low impedance values for skin contact [28,29]. However, the contact impedance of the semi-dry electrode is slightly higher than that of the wet electrode. Despite this, the semi-dry electrode proposed in this study is more comfortable and features an “on/off” capability, allowing it to control the release of conductive fluid artificially, making it more suitable for various settings.

#### 3.1.2. Service Life of Electrodes

**Figure 7:**
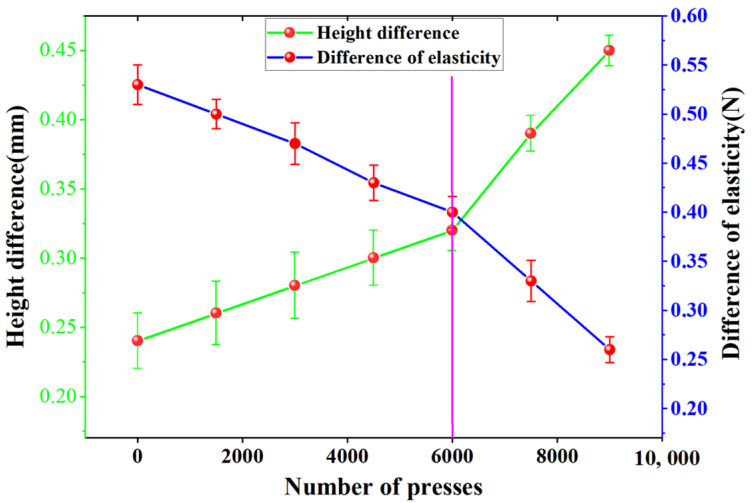
Curve of the mechanical life of the electrode.

The pressure between the skin and the semi-dry electrode can affect the quality of the obtained signal [30]. However, the elasticity of the spring inside the electrode gradually decreases with prolonged use of the electrode, which affects the obtained signal quality. Consequently, mechanical life testing of the electrodes is required. Figure 7 displays the service life curve of the semi-dry electrode proposed in this research.

As depicted in Figure 7, the decrease in elasticity and increase in height difference in the electrode is relatively slow until the number of presses reaches 6000. However, once 6000 presses are reached, the decrease in electrode elasticity and increase in height difference is rapid. This suggests that after 6000 presses, the electrode will show a substantial decrease in performance.

#### 3.1.3. Long-Term Stability Comparison

**Figure 8:**
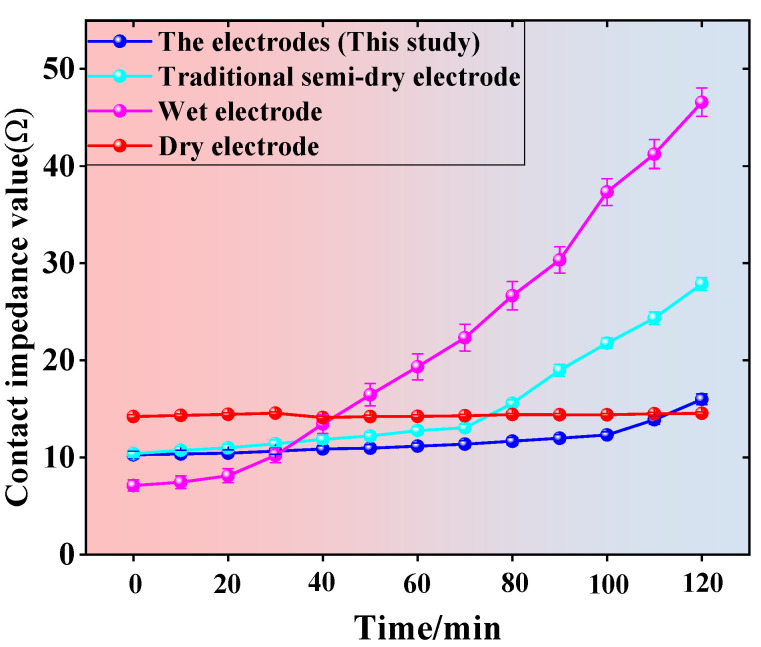
Long-term stability test experiment conducted in this study.

This study conducted stability tests on EEG signal acquisition using electrodes. Considering that traditional wet electrodes require the removal and reapplication of conductive gel after approximately two hours of continuous use to continue EEG signal acquisition, this study selected new semi-dry electrodes, traditional wet electrodes, and traditional dry electrodes to conduct a two-hour EEG signal acquisition experiment, ensuring no interruptions during the acquisition process. During the experiment, the impedance changes between the electrodes and the scalp were measured every 10 min. The impedance data for the four types of electrodes are shown in Figure 8, which clearly illustrate the performance of different electrode types during long-term use.

Figure 8 shows that when using standard wet electrodes, the conductive paste gradually evaporates over time, which leads to a gradual increase in contact impedance between the electrode and the skin. In contrast, although the impedance of conventional semi-dry electrodes also increases over time, the increase is smaller than that of wet electrodes. The new semi-dry electrode has lower volatility of its conductive liquid, which results in a significantly smaller increase in impedance compared to traditional wet electrodes and conventional semi-dry electrodes. For dry electrodes, since there is no conductive liquid between the electrode and the skin, the effects of conductive liquid evaporation are avoided, resulting in minimal changes in impedance over different time periods. However, due to the absence of conductive liquid, the contact impedance between the dry electrode and the skin remains consistently high.

### 3.2. MF-DFA

#### 3.2.1. The Fluctuation Function

**Figure 9:**
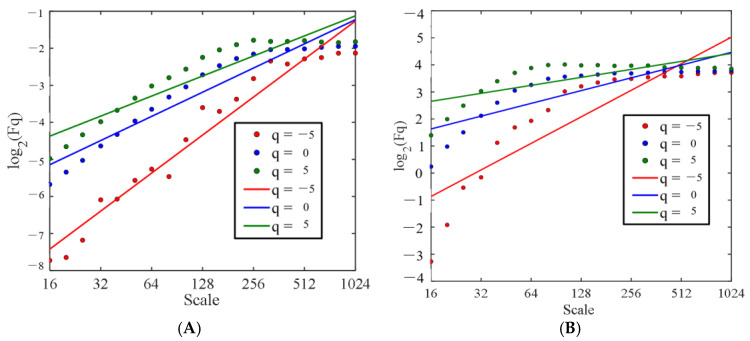
Fluctuation functions of the O1 channel EEG signals recorded with the semi-dry electrodes. (**A**) Theta waves; (**B**) beta waves.

**Figure 10:**
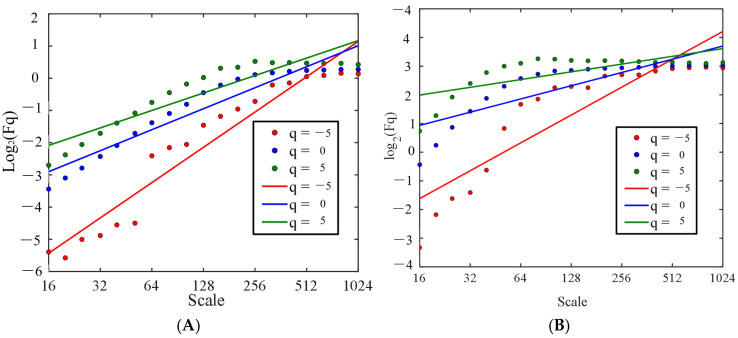
Fluctuation functions of the O1 channel EEG signals recorded with the Emotiv electrode. (**A**) Theta waves; (**B**) beta waves.

The fluctuation function can characterize the volatility and scale of the signal [31]. Figure 9 and Figure 10 display the fluctuation functions plots of the theta and beta waves of the O1 channel EEG signal during stage 1 of subject A. The data were taken at various orders (q) and time scales using two electrodes.

The double logarithmic relationship between the change in scale s for order q and the mean square error function Fq(s) of the EEG signal is depicted in Figure 9 and Figure 10. The Hurst exponent is the slope of a linear fit between the mean squared error function Fq(s) and the logarithm of scale s. The slope of the curve fitted using the least squares method based on the log values of Fq(s) and scale s is known as the Hurst exponent. log Fq(s) has a good linear relationship with the logarithm of scale; that is, the relationship between Fq(s) and s follows a power law. EEG signals exhibit scale invariance at a certain scale, which is a typical geometric property of the multifractal spectrum and can be studied using multifractal theory [32].

#### 3.2.2. Mass Exponent

**Figure 11:**
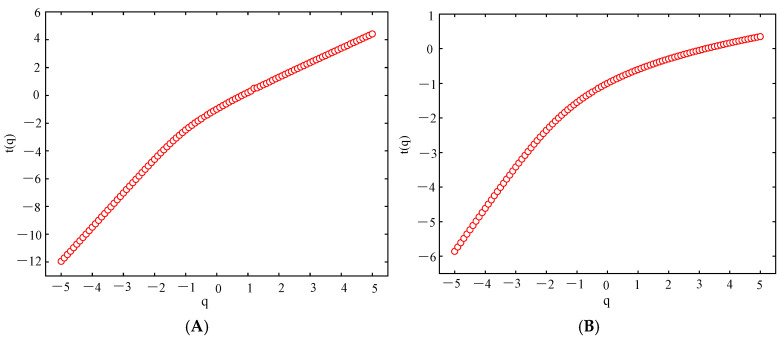
Mass exponent curve of the EEG signal acquired from the semi-dry electrodes in the O1 channel. (**A**) Theta waves; (**B**) beta waves.

**Figure 12:**
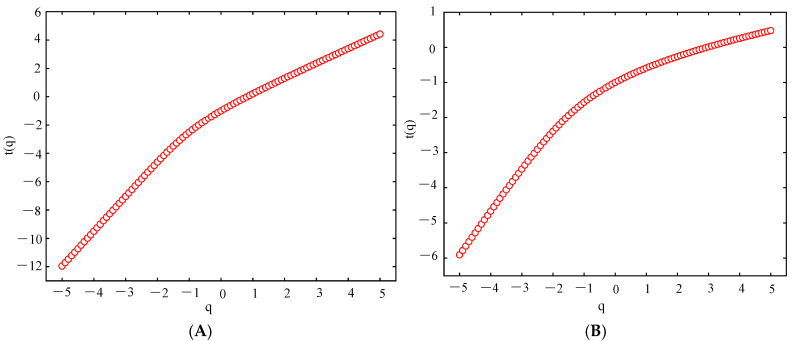
Mass exponent curve of the EEG signal acquired from the Emotiv electrode in the O1 channel. (**A**) Theta waves; (**B**) beta waves.

Figure 11 and Figure 12 show the mass exponent plots of theta and beta waves at different orders (q) of the O1 channel EEG signal of stage 1 of subject A acquired by the two electrodes.

Figure 11 and Figure 12 show the variation in the mass exponent τ(q) with the fractal order q. The curvature of the mass exponent curve can be used to depict the multifractal nature of the signal. The greater the curvature, the more pronounced the multifractal characteristics [33]. The figure shows that the mass exponent has a nonlinear relationship with q, and the EEG signals exhibit multifractal characteristics. Therefore, EEG signals can be analyzed using the MF-DFA method.

#### 3.2.3. Hurst Exponent

**Figure 13:**
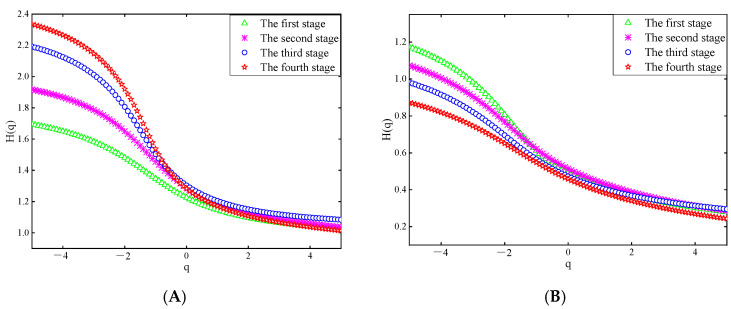
Hurst exponent of the EEG signal recorded by the semi-dry electrode. (**A**) Theta waves; (**B**) beta waves.

**Figure 14:**
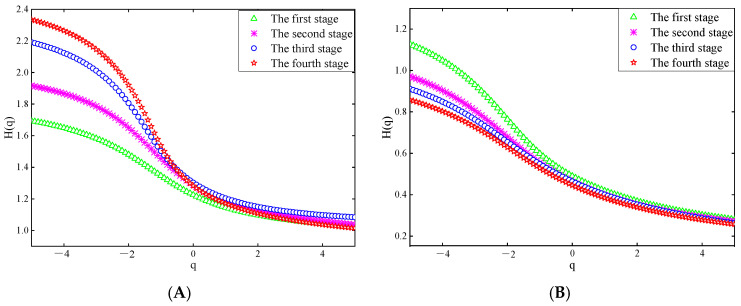
Hurst exponent of EEG signals recorded Emotiv electrodes. (**A**) Theta waves; (**B**) beta waves.

Figure 13 and Figure 14 show the Hurst exponents of the theta and beta waves of subject A’s O1 channel EEG data, obtained by the two electrodes, respectively.

Figure 13 and Figure 14 show that when q is less than 0, the Hurst exponents of each driving stage are clearly distinguished. With the increase in q, the rate at which the value of the Hurst exponent decreases first increases and then decreases, which indicates that the fractal characteristics of EEG signals show certain inhomogeneity [34]. Specifically, it can be seen from Figure 13A and Figure 14A that in the theta waves of the EEG signals collected by the two electrodes, the range of the Hurst exponent gradually expands as the driving phase increases. However, in Figure 13B and Figure 14B, the range of the Hurst exponent decreases gradually for the beta waves. It has been shown that a larger range of Hurst exponents of a signal indicates a higher complexity. The complexity of the theta waves in the EEG signals obtained by the two electrodes increases gradually, while the complexity of the beta waves diminishes gradually, as observed in Figure 13 and Figure 14. This is consistent with the changing characteristics of theta waves and beta waves when human mental fatigue occurs.

#### 3.2.4. Multifractal Spectrum

**Figure 15:**
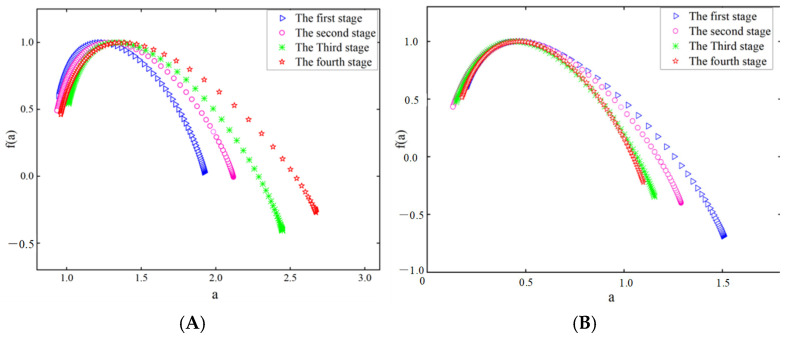
Multifractal spectral width of EEG signals recorded by the semi-dry electrode. (**A**) Theta waves; (**B**) beta waves.

**Figure 16:**
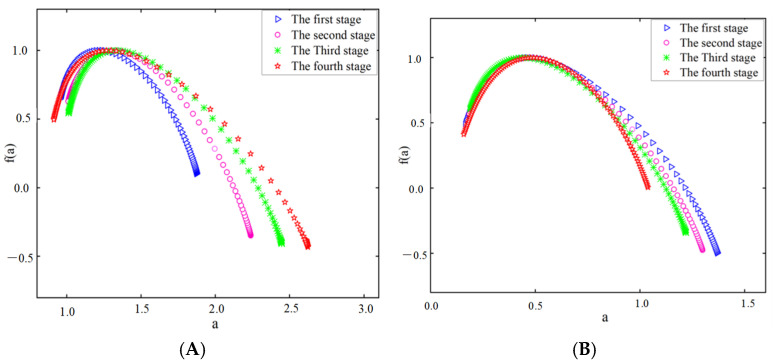
Multifractal spectral width of EEG signals recorded by Emotiv electrodes. (**A**) Theta waves; (**B**) beta waves.

Figure 15 and Figure 16 show the multifractal spectra of the theta and beta waves of subject A’s O1 channel EEG data, obtained by the two electrodes, respectively.

The multifractal spectrum of EEG signals exhibits a unimodal shape, as indicated by the results in Figure 15 and Figure 16. A broader spectrum shows a stronger fractal quality, suggesting that the EEG signal exhibits multifractal properties and is categorized as chaotic signals [35]. Distinct variations are observed in the multifractal spectra at different driving stages. The width of the multifractal spectrum of the theta waves in the EEG data obtained by the two electrodes increased as the driving stage progressed, while the width of the multifractal spectrum of the beta waves decreased. The width of the multifractal spectrum indicates the complexity and magnitude of fluctuations in EEG signals during the entire procedure. Thus, as the fatigue level increases, the intricacy of the theta waves will increase, while the complexity of the beta waves will decrease for the subject.

#### 3.2.5. Statistical Analysis

**Figure 17:**
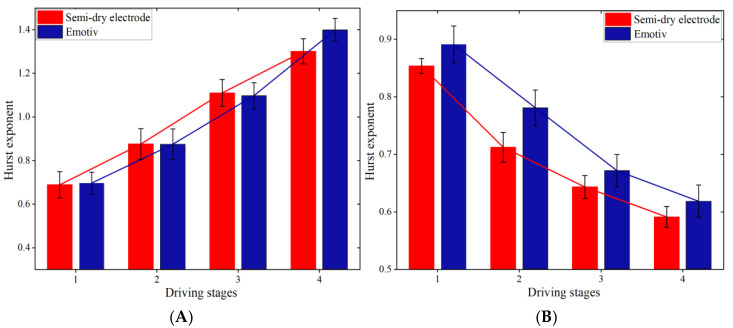
The average Hurst exponent range of O1 channel EEG signals of 12 subjects recorded using two electrodes. (**A**) Theta waves; (**B**) beta waves.

**Figure 18:**
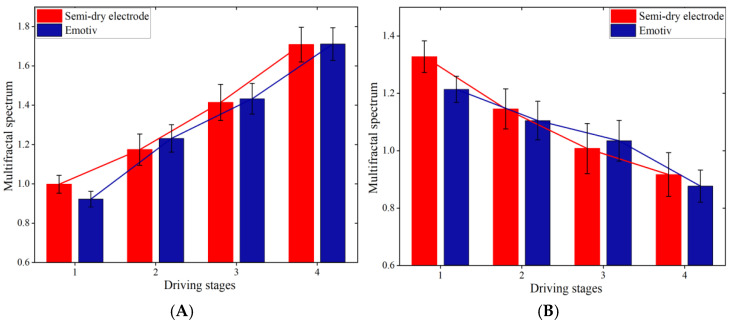
The average multifractal spectral width of O1 channel EEG signals of 12 subjects recorded using two electrodes. (**A**) Theta waves; (**B**) beta waves.

In this study, the Hurst exponent range and multifractal spectrum width of 12 subjects in four driving stages were counted, as depicted in Figure 17 and Figure 18.

Research indicates that as mental tiredness escalates, theta wave activity in the EEG signal increases, while beta wave activity decreases. Figure 17 and Figure 18 show that the average Hurst exponent and multifractal spectrum width of the theta waves in the O1 channel EEG signal continuously increase, while the average Hurst exponent and multifractal spectrum width of the beta waves continuously decrease. This suggests that the complexity of theta waves steadily increases, while the complexity of beta waves steadily decreases as driving time increases. This is consistent with earlier research [36]. Therefore, the MF-DFA method proposed in this paper effectively detects and quantifies mental fatigue in crane operators.

### 3.3. Correlation Analysis

The application of wet electrodes is prevalent in scientific, cognitive, and clinical research [37]. As a result, it is essential to analyze the correlation between the EEG signals collected in this investigation utilizing wet and semi-dry electrodes.

**Figure 19:**
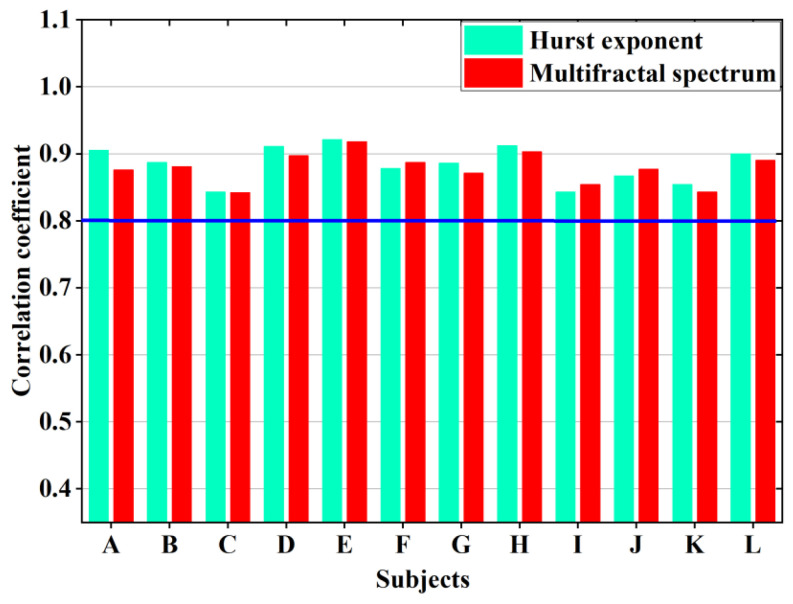
Correlation coefficients of fatigue characteristic parameters between the two types of electrodes.

Figure 19 illustrates that all of the correlation coefficients between the semi-dry electrode and the wet electrode proposed in this work are over 0.8, indicating a highly substantial link between the aspects of weariness of the EEG signals obtained by the two electrodes. The impact of the semi-dry electrode and the wet electrode on signal acquisition is almost the same.

## 4. Discussion

### 4.1. CS Method

This study used the CS method to preprocess EEG signals, and the results are shown in Table 1.

According to the comparative data of the application effects of compressive sensing technology provided in Table 1, it is clear that this method significantly improves the training and inference efficiency of the model. Regarding training time, after adopting the compressive sensing method, the training time of the model was reduced from 32.67 s to 24.34 s, an absolute reduction of 8.33 s, corresponding to a relative efficiency improvement of 25.5%. This indicates that compressive sensing reduces computational complexity through sparsification, thus accelerating model convergence. Regarding testing speed, the time required for a single training session decreased from 17.33 ms to 14.68 ms, an absolute reduction of 2.65 ms, leading to a relative speedup of 15.3%.

### 4.2. Rotary Switch-Type Semi-Dry Electrode

Traditional wet electrodes are mostly designed for subjects in static states, such as sitting or lying. Although the conductive paste of wet electrodes reduces the contact impedance and improves the quality of collected signals, it is time-consuming and laborious to apply, and it degrades over time. In severe cases, it can even cause skin allergies. In addition, wet electrodes require shaving or trimming the scalp before use, which requires professional operation, high time and operational complexity, and provides low comfort for the subjects. The hair needs to be cleaned after the experiment. These limitations significantly restrict the use of wet electrodes in wearable EEG signal collection sensors. In future applications of brain–computer interfaces, wearable EEG acquisition systems may be widely used [38]. To solve the problems caused by the use of conductive paste, such as cumbersome operation and difficulty in maintaining for long periods, dry electrodes were first invented in the 1990s.

Dry electrodes require less preparation time than wet electrodes before use and do not require skin preparation. Dry electrodes do not require conductive paste, can be used immediately, significantly shortening the placement time, and are suitable for home testing. There is also little need to clean up after use. In some cases, they can be used without training. However, it is difficult to adhere dry electrodes to the scalp. The significant signal instability and high impedance make them more susceptible to power interference and motion artifacts compared to wet electrodes [39]. In recent research on EEG signal collection technologies, semi-dry electrodes have drawn a lot of attention due to their greater comfort, stability, and convenience compared to conventional wet and dry electrodes. However, all of the current semi-dry electrodes share the same flaw: they are unable to regulate the outflow of conductive liquid [40]. By designing a rotating switch structure, the semi-dry electrode proposed in this study can turn off when not in use, preventing the outflow of conductive liquid. When used, the “switch” is turned on, allowing the conductive liquid to flow out and make contact with the skin. The rotating “switch” structure designed in this study can significantly improve the utilization efficiency of the conductive solution, extending the use time of the electrode, which meets the needs of a wider range of experimental settings and prevents the erosion of the skin by excessive conductive solution when EEG signals are not needed.

In addition, electrode fabrication materials used for EEG signal acquisition usually include pure metals (such as gold, silver, platinum, copper, etc.) and silver/silver chloride [41]. Electrodes made of pure metal often have disadvantages, such as poor contact stability and low comfort due to their greater rigidity. Cylindrical metal electrodes usually need to apply specific pressure to make contact with the skin, which may easily cause skin discomfort or infection [42]. These materials may cause discomfort for the subjects. Therefore, this study chose to use conductive sponges instead of rigid conductive materials. Compared with hard conductive materials, conductive sponges cannot only collect high-quality EEG signals during use but also provide better comfort, and they are not easily prone to skin irritation or wear, which helps to reduce the discomfort of wearers and prolong wearing time.

The comparison between the electrode proposed in this study and other electrodes is shown in Table 2.

Table 2 shows that while the traditional wet electrode has the lowest contact impedance compared to other electrode types, it can only be used for up to 2 h continuously. Dry electrodes can be used constantly without the need for the frequent addition of conducting fluid, which is not the case with wet electrodes. However, the contact impedance value between the electrodes and the scalp is high, resulting in a decrease in the quality of the acquired signal. The semi-dry electrode offers the advantages of both wet and dry electrodes, resulting in a reduced impedance value and an extended continuous usage time. In comparison with other semi-dry electrodes, the rotary switch-type semi-dry electrode suggested in this investigation exhibits the longest duration for continuously collecting the signal and possesses the lowest impedance value.

### 4.3. Fatigue Detection Method

**Figure 20:**
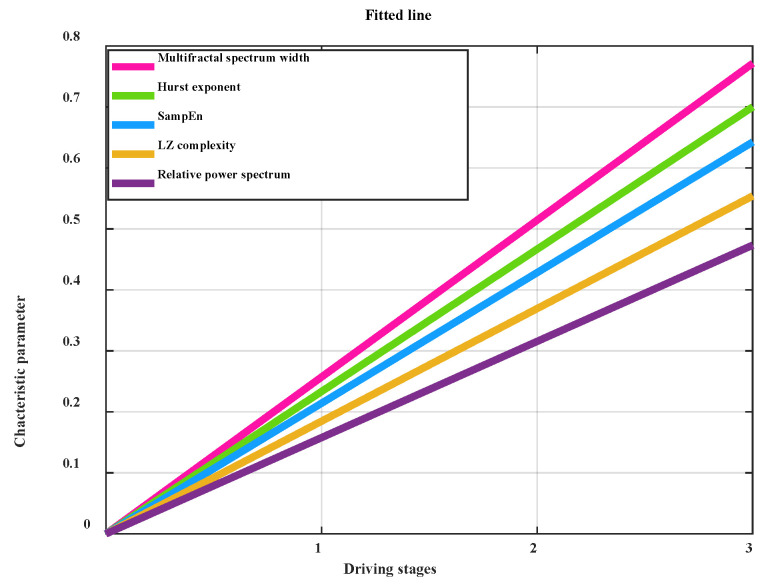
Comparing the slope of the fitted line using different approaches.

Traditional approaches to driving fatigue have some limitations. For example, the SampEn method is sensitive to noise, and if noise is present in the signal, it may cause disturbances in the calculation results, resulting in a decrease in accuracy [47]. The power spectral approach, which transforms the signal from the time domain to the frequency domain, may introduce a certain amount of time–frequency uncertainty. At different frequencies, there is a trade-off between the time resolution and frequency resolution of the signal, meaning that the instantaneous time domain and frequency domain information of the signal cannot be captured simultaneously [48]. LZ complexity is highly influenced by the signal’s length, primarily reflecting the overall features of the signal, and showing less sensitivity to its local properties [49]. To address these limitations of conventional methods, the MF-DFA method was employed in this study to identify mental exhaustion in crane operators. The MF-DFA method can effectively capture nonlinear features in EEG signals, including long-range correlations and fractal features, which are crucial for understanding the dynamic processes of brain activity. Unlike some traditional methods, the MF-DFA method does not require stationarity assumptions on the signal, making it suitable for non-stationary signals with complex dynamic characteristics. Moreover, the MF-DFA method demonstrates good adaptability in both local and global feature analysis, considering both long-range and short-range fluctuations of the signal. Additionally, MF-DFA is more effective in capturing the dynamic characteristics of a signal, as it is more resistant to noise and can partially counteract its impact [50]. To demonstrate that the MF-DFA method adopted in this study can better extract fatigue features, we extracted fatigue features based on the theta waves of the O1 channel using SampEn, LZ complexity, and relative power spectrum methods, respectively. The fitted lines and their slopes for these features in the four driving stages are presented in Figure 20.

The slope values of the fitted line for the multifractal spectrum width and Hurst exponent are the highest in the four driving stages, as shown in Figure 20. The MF-DFA approach used in this study demonstrates the maximum sensitivity in collecting fatigue features, making it more effective in reflecting the trend of mental fatigue changes in crane operators.

### 4.4. Limitations and Future Prospects

The suggested rotary switch-type semi-dry electrode has a complicated mechanical structure on the inside, which makes it hard to clean and keep up. Furthermore, making a rotary switch-type semi-dry electrode might cost more than making a regular electrode.

## 5. Conclusions

This study proposes a rotating switch-type semi-dry electrode for collecting EEG signals from crane operators, followed by the use of the MF-DFA method to detect the mental fatigue state of crane operators. The main conclusions are as follows:

### 5.1. The Rotating Switch-Type Semi-Dry Electrode

This study presents a rotating switch-type semi-dry electrode that achieves significant breakthroughs in key performance indicators. First, the electrode–skin contact impedance at 10 Hz is 12.3 kΩ, approximately 26% lower than that of traditional dry electrodes (16.7–17.4 kΩ), approaching the performance of wet electrodes (9.5 kΩ), and significantly outperforming existing semi-dry electrodes (13.8–14.6 kΩ). Second, the electrode’s service life has been significantly extended: by precisely controlling the release of conductive liquid using the rotating switch structure, the electrode can operate continuously for up to 10 h, a fivefold increase compared to traditional wet electrodes (2 h) and a 25% improvement over the best-performing semi-dry electrodes in the same category (8 h). Finally, the mechanical stability has been rigorously validated: after 6000 compression tests, the electrode’s elasticity decreased by only 15%, and the height deformation was less than 0.5 mm, fully meeting the mechanical reliability requirements for long-term wear.

### 5.2. The MF-DFA Methods

This study employed the MF-DFA method to achieve the precise quantification of crane operators’ mental fatigue states. This method demonstrates strong sensitivity to fatigue features, specifically within the Hurst index range of θ waves, with a 42% increase observed during a 4 h driving task. At the same time, the multifractal spectral width of β waves decreased by 38%. Furthermore, the method’s superiority was confirmed: the MF-DFA’s fitting slope for fatigue features reached 0.32, indicating a 44–78% improvement over sample entropy and LZ complexity, highlighting its superior ability to capture nonlinear EEG features. Concerning signal consistency, the correlation coefficient between fatigue features collected using semi-dry electrodes and wet electrodes reached 0.92, further validating its signal reliability in real construction site environments.

## Figures and Tables

**Table 1:** Comparison of training times with and without compressive sensing.

Method	Training Time (s)	Test Time (ms)
Not using CS methods	32.67	17.33
Using CS methods	24.34	14.68

**Table 2:** Comparison of electrode performance.

Types of Electrodes	The Amount of Conductive Liquid	Impedance (10 Hz)	Hours of Use
Ag/AgCl wet electrode	Need more	9.5 KΩ	2 h
Emotiv electrode	Need more	10.6 KΩ	2 h
A novel dry-contact electrode [43]	No need	16.7 KΩ	/
Passive dry electrodes [44]	No need	17.4 KΩ	/
Flexible multilayer semi-dry electrode [14]	Need less	14.6 KΩ	5 h
Novel superporous hydrogel-based semi-dry EEG electrodes [45]	Need less	13.8 KΩ	8 h
Novel semi-dry electrode [11]	Need less	13.8 KΩ	8 h
Semi-dry electrode [46]	Need less	14.1 KΩ	8 h
Rotary switch-type semi-dry electrode (this study)	Need less	12.3 KΩ	10 h

## Data Availability

All data included in this study are available upon request by contact with the corresponding author. The data are not publicly available because of ethical restrictions.
